# Growth-Promoting and Quality-Enhancing Effects of Insect-Derived *Serratia marcescens* BRC-CXG2 on Romaine Lettuce

**DOI:** 10.3390/ijms27073136

**Published:** 2026-03-30

**Authors:** Xinran Hu, Yukun Zhu, Zhao Wu, Guoxi Ji, Zhitong Lin, Moyan Wang, Fen Li, Jiaping Xu, Kaiqi Wu, Wenyu Tian, Xiaohong Han

**Affiliations:** 1 College of Tea and Food, Wuyi University, Wuyishan 354300, China; 18208368769@163.com (X.H.); wuzhao@wuyiu.edu.cn (Z.W.); 18959807729@163.com (Z.L.); 15359960379@163.com (M.W.); 18685520846@163.com (F.L.); xujiaping-study@outlook.com (J.X.); m17744062854@163.com (K.W.); m17390543006@163.com (W.T.); 2 State Key Laboratory of Agricultural and Forestry Biosecurity, College of Forestry, Fujian Agriculture and Forestry University, Fuzhou 350002, China; zhuyukun001@126.com (Y.Z.); jgxybyqkkk@163.com (G.J.)

**Keywords:** *Serratia marcescens* BRC-CXG2, plant growth-promoting bacteria, romaine lettuce, biomass accumulation, rhizosphere microbial community

## Abstract

To explore the application potential of insect-derived functional microorganisms in short-cycle leafy vegetable production, we evaluated the effects of *Serratia marcescens* BRC-CXG2, isolated from larvae of *Monochamus alternatus*, on romaine lettuce in a pot experiment. Plant growth traits, biomass accumulation, nutritional quality, endogenous hormones, and rhizosphere microbial communities were systematically evaluated. The results demonstrated that inoculation significantly promoted seedling development. Plant height and root length increased by 48.7% and 29.1%, respectively, while shoot and root dry weights were 1.78- and 1.85-fold higher than those of the control. Vitamin C and total sugar contents increased by 76.4% and 98%, respectively. The levels of gibberellins (GA_3_)-, indole-3-acetic acid (IAA)-, and abscisic acid (ABA)-immunoreactive equivalents increased by 1.5-, 1.29-, and 1.75-fold. High-throughput 16S rDNA gene and ITS amplicon sequencing further revealed that inoculation reshaped the composition of bacterial and fungal communities in the rhizosphere. Collectively, these findings demonstrate that insect-derived *S. marcescens* exhibits significant growth-promoting potential in short-cycle leafy vegetable systems, with effects associated with hormone regulation, enhanced total sugar accumulation, and shifts in rhizosphere microbial community structure.

## 1. Introduction

With the advancement of sustainable agriculture, developing safe and effective plant growth-promoting strategies has become a key priority in vegetable production. Plant growth-promoting rhizobacteria (PGPR) enhance nutrient uptake, regulate endogenous hormone levels, and promote coordinated root and shoot development, thereby reducing reliance on chemical fertilizers and improving crop productivity [1,2,3]. While the application potential of PGPR has been well established in grain and certain economic crops, their application in short-cycle leafy vegetables remains limited [4].

*Serratia marcescens*, a Gram-negative bacterium with strong ecological adaptability, is widely distributed in soil, plants, and insects [5]. In recent years, increasing attention has been paid to its plant growth-promoting potential. Numerous studies have shown that it can promote plant growth through multiple mechanisms, including the production of indole-3-acetic acid (IAA) [6,7], phosphate solubilization [8,9,10], the secretion of bioactive metabolites [11,12,13], and the modulation of rhizosphere microbial community structure [14,15]. Based on this, *S. marcescens* is considered a rhizosphere bacterium with potential applications, showing growth-promoting effects in crop systems such as cucumber, pepper, rice, and maize. Notably, most reported *S. marcescens* strains with plant growth-promoting functions have been isolated from soil or plant endophytic environments, whereas studies on strains isolated from insects remain limited. In fact, insects represent a specific ecological niche with dynamic nutrient availability and significant immune pressure, in which microorganisms that establish long-term colonization typically exhibit stronger environmental adaptability and host interaction capabilities [16,17]. Previous studies have shown that *S. marcescens* isolated from insects can stably colonize rice and significantly promote plant growth [18], indicating that insect-derived *S. marcescens* may represent an underexplored resource for plant growth promotion. However, to date, relevant studies have been primarily focused on rice and other grain crops, and the application potential of *S. marcescens* in short-cycle leafy vegetables, which are highly sensitive to nutrient availability and hormone levels, has not yet been systematically evaluated.

Romaine lettuce (*Lactuca sativa* var. *longifolia*) is an important leafy vegetable that is widely cultivated in vegetable production and has stable consumer demand. It has a short growth cycle and shows a rapid response to exogenous regulation [19], making it a suitable model for evaluating the growth-promoting effects of microorganisms. Systematically evaluating the growth-promoting effects of insect-derived *S. marcescens* in this system can contribute to a more comprehensive understanding of its application value in short-cycle vegetable production and provide a scientific basis for its subsequent development and utilization. Based on this, the present study used a strain of *S. marcescens* isolated from *Monochamus alternatus* as the research object. Through pot inoculation experiments, it systematically analyzed its effects on plant growth traits, biomass accumulation, nutritional quality, and endogenous hormone levels of romaine lettuce, together with changes in the rhizosphere microbial community structure, to evaluate its growth-promoting effects and potential underlying mechanisms in short-cycle leafy vegetable systems.

## 2. Results

### 2.1. Growth and Physiological Parameters of Romaine Lettuce

#### 2.1.1. Seedling Growth and Phenotype of Romaine Lettuce

**Figure 1:**
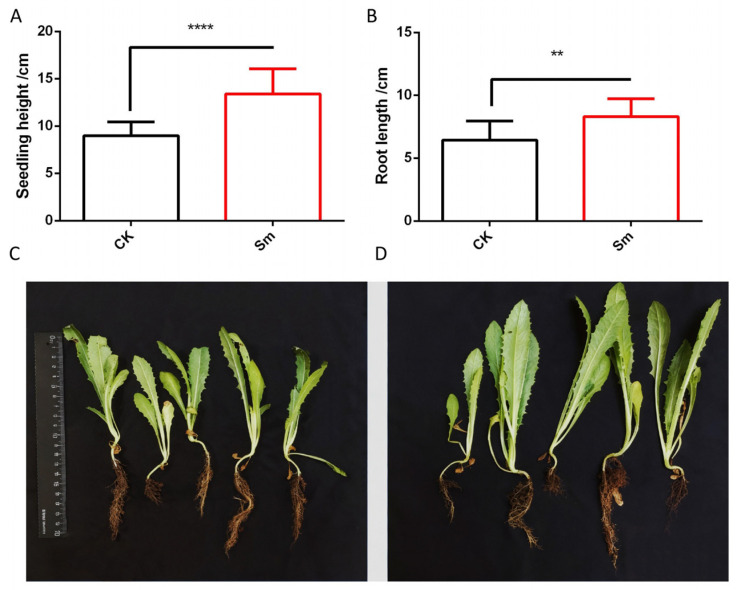
Effects of Sm treatment on seedling height, root length, and growth phenotype of romaine lettuce. (**A**) Plant height of seedlings in the control and Sm-treated groups; (**B**) Root length of seedlings in the control and Sm-treated groups; (**C**) Phenotype of control seedlings; (**D**) Phenotype of Sm-treated seedlings. Data are presented as mean ± standard deviation. ** indicates *p* < 0.01, **** indicates *p* < 0.0001, *n* = 14.

Compared with the control, the seedlings treated with *S. marcescens* BRC-CXG2 (Sm) showed significant increases in plant height and root length (*p* < 0.0001). Specifically, the average plant height of seedlings in the control (CK) group was 8.99 ± 1.44 cm, whereas it increased to 13.38 ± 2.67 cm in the Sm-treated group, representing a 48.7% increase. For root length, the average root length of CK seedlings was 6.44 ± 1.52 cm, which increased to 8.32 ± 1.41 cm after Sm treatment, corresponding to an approximate 29.1% increase, indicating that Sm treatment promoted both shoot elongation and root development (Figure 1A,B).

As shown in the phenotypic images of seedlings in Figure 1C (CK group) and Figure 1D (Sm-treated group), the overall growth of CK seedlings was weaker, with shorter plant height, less developed roots, and smaller leaves, whereas Sm-treated seedlings exhibited more vigorous growth, with greater plant height, more developed and elongated roots, and larger, broader leaves, providing direct visual evidence that Sm treatment promoted both aboveground and belowground growth of the seedlings.

#### 2.1.2. Determination of Fresh and Dry Biomass of Romaine Lettuce

**Figure 2:**
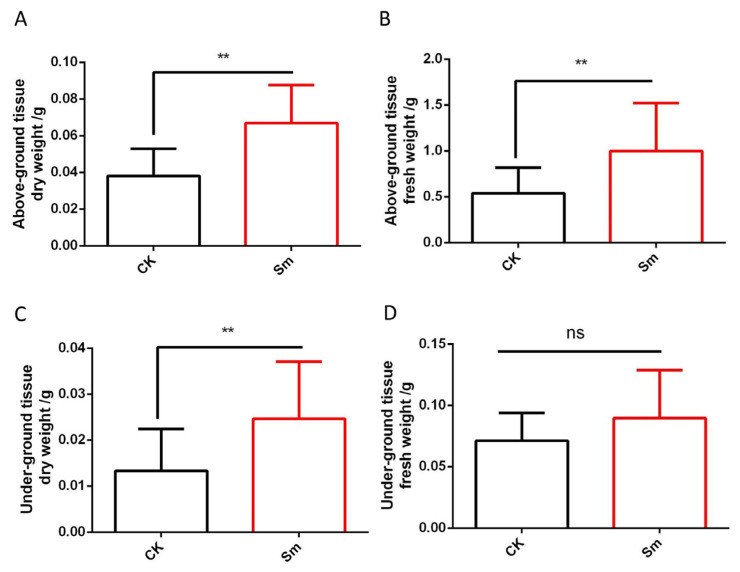
Effects of Sm treatment on the dry and fresh biomass of the aboveground and belowground parts of romaine lettuce. (**A**) Aboveground dry biomass; (**B**) Aboveground fresh biomass; (**C**) Belowground dry biomass; (**D**) Belowground fresh biomass. CK represents the control group, and Sm represents the Sm-treated group. Data are presented as mean ± standard deviation. ** indicates *p* < 0.01, ns indicates no significant difference, *n* = 15.

This study analyzed the effects of Sm treatment on the fresh and dry biomass of the aboveground and belowground parts of the plants. As illustrated in Figure 2A, the aboveground dry biomass of the Sm-treated group was significantly higher than that of the control, with CK seedlings measuring 0.038 ± 0.015 g and Sm-treated seedlings increasing to 0.067 ± 0.021 g, representing an approximately 76.1% increase. Similarly, the aboveground fresh weight in the Sm-treated group was significantly higher than that in the CK (Figure 2B). For the belowground dry biomass (Figure 2C), the Sm-treated group was significantly higher than the CK group, with CK seedlings at 0.013 ± 0.01 g and Sm-treated seedlings at 0.025 ± 0.013 g, corresponding to an approximately 85% increase, indicating that Sm treatment promoted root dry matter accumulation. Regarding belowground fresh biomass (Figure 2D), CK and Sm-treated seedlings measured 0.071 ± 0.023 g and 0.090 ± 0.039 g, respectively. Although there was an approximate 25.8% increase, the difference was not statistically significant, suggesting that Sm treatment had no significant effect on belowground fresh biomass.

### 2.2. Measurement of Nutritional Quality of Romaine Lettuce

**Figure 3:**
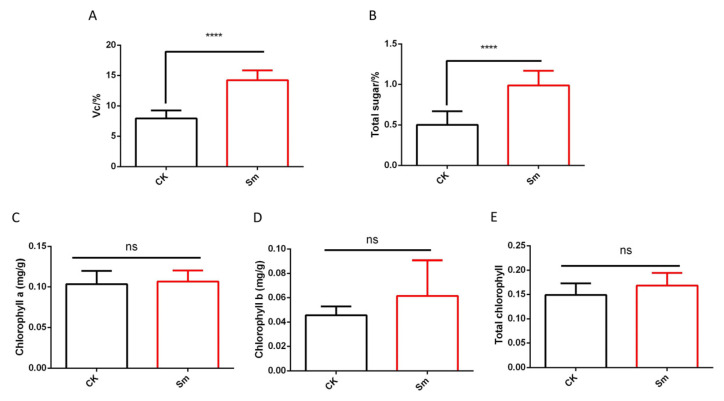
Effects of Sm treatment on vitamin C, total sugar, and chlorophyll content in romaine lettuce. (**A**) Vitamin C content; (**B**) Total sugar content; (**C**) Chlorophyll a content; (**D**) Chlorophyll b content; (**E**) Total chlorophyll content. Data are presented as mean ± standard error. **** indicates *p* < 0.0001; ns indicates no significant difference.

This study systematically investigated the effects of Sm treatment on multiple physiological parameters of the plant, including vitamin C content, total sugar content, and chlorophyll components (chlorophyll a, chlorophyll b, and total chlorophyll), to elucidate the regulatory effects of Sm on plant physiological metabolism. Vitamin C, as an important antioxidant and nutritional component in plants, is directly associated with plant physiological status and nutritional quality. As shown in Figure 3A, Sm treatment significantly increased the vitamin C content in romaine lettuce. The vitamin C content in the CK group was 8.06 ± 0.40, whereas it increased to 14.22 ± 0.52 in the Sm-treated group, representing an increase of approximately 76.4% compared with the control. This result suggests that Sm treatment promoted the synthesis and accumulation of vitamin C in the plants. The increased vitamin C content may enhance plant antioxidant capacity and tolerance to oxidative stress, as well as improve nutritional quality. It therefore plays a crucial role in plant physiological metabolism and quality formation, reflecting the positive regulatory effect of Sm treatment on plant nutritional physiology.

Total sugars are important energy sources and metabolic intermediates in plants, and changes in their content can reflect the level of carbon metabolism. Figure 3B shows that the total sugar content in the Sm-treated group was significantly higher than that in the CK group. The CK group exhibited a value of 0.50 ± 0.05, whereas the Sm-treated group reached 0.99 ± 0.06, representing an increase of approximately 98%. These results suggest that Sm treatment promoted the accumulation of carbon assimilation products in the plants. Chlorophyll is the core pigment involved in photosynthesis in plants, and its content and composition are directly related to the efficiency of light energy capture and conversion. Statistical analysis showed that there were no significant differences between the Sm-treated group and the CK group in the contents of chlorophyll a (Figure 3C), chlorophyll b (Figure 3D), or total chlorophyll (Figure 3E). These results indicate that Sm treatment did not significantly affect chlorophyll biosynthesis or accumulation, and the photosynthetic pigment system remained relatively stable.

### 2.3. Determination of Hormone Levels in Romaine Lettuce

**Figure 4:**
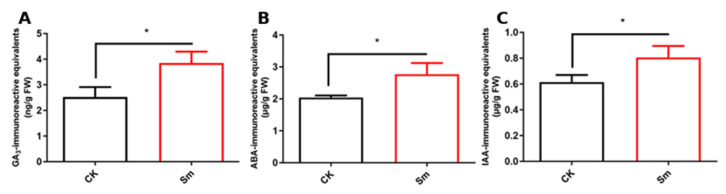
Effects of Sm treatment on hormone contents in romaine lettuce. (**A**) GA_3_-immunoreactive equivalents; (**B**) ABA-immunoreactive equivalents; (**C**) IAA-immunoreactive equivalents. Data are presented as mean ± standard error. * indicates *p* < 0.05, *n* = 3.

To investigate the effects of Sm treatment on hormone levels in romaine lettuce, the contents of gibberellins (GA)-, abscisic acid (ABA)-, and indole-3-acetic acid (IAA)-immunoreactive equivalents were measured following Sm treatment (Figure 4). The levels of GA_3_-, ABA-, and IAA-immunoreactive equivalents increased significantly by 1.5-, 1.75-, and 1.29-fold, respectively (*p* < 0.05). These results suggest that Sm treatment elevated hormone levels in the plants, which may contribute to the regulation of plant physiological processes.

### 2.4. Comparison of Microbial Diversity

#### 2.4.1. Principal Coordinates Analysis (PCoA) of Samples

**Figure 5:**
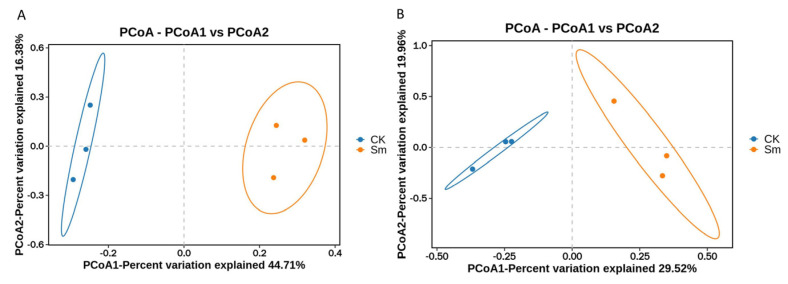
Differences in community structure of samples based on Principal Coordinates Analysis (PCoA). (**A**) PCoA plot of the main experimental dataset (PCoA1: 44.71%; PCoA2: 16.38%); (**B**) PCoA plot of the independent validation dataset (PCoA1: 29.52%; PCoA2: 19.96%). Each point represents a sample, and colors indicate treatment groups: blue, CK control; orange, Sm-treated.

The samples from the CK and Sm groups exhibited clear clustering separation in the two-dimensional space, with no overlapping distribution between the two groups (Figure 5A). CK and Sm samples clustered in distinct regions, and within-group dispersion was relatively low (Figure 5B), indicating good reproducibility and a stable effect of Sm treatment. These results suggest that Sm treatment significantly reshaped the overall characteristics of the samples, with clear differences observed between the two groups.

#### 2.4.2. Relative Abundance of Communities at Different Taxonomic Levels Under Sm Treatment

**Figure 6:**
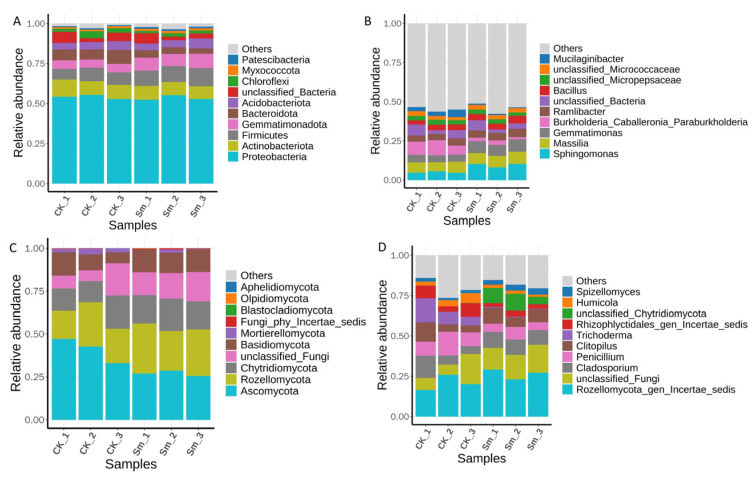
Relative abundances of microbial communities at different taxonomic levels. (**A**) Relative abundances at the bacterial phylum level; (**B**) Relative abundances at the bacterial genus level; (**C**) Relative abundances at the fungal phylum level; (**D**) Relative abundances at the fungal genus level.

To elucidate the effects of Sm treatment on microbial community composition, the relative abundances of communities at different taxonomic levels (phylum and genus) were analyzed for the control (CK) and Sm-treated groups (Figure 6). The bacterial community was first analyzed. In the control group, the dominant phylum was Proteobacteria. Following Sm treatment, Proteobacteria remained dominant, while the relative abundance of Gemmatiomonadota increased and Bacteroidota decreased (Figure 6A). At the genus level, bacterial community composition differed between the control and Sm-treated groups. The relative abundances of *Gemmatimonas* and *Sphingomonas* were higher in the Sm-treated samples, whereas *Mucilaginibacter* and the *Burkholderia_Caballeronia_Paraburkholderia* group were more abundant in the control group. (Figure 6B).

The fungal community in the control group was dominated by the phylum *Ascomycota*, which accounted for the absolute majority. After Sm treatment, the abundance of *Ascomycota* slightly decreased (Figure 6C). At the genus level, fungal community composition also differed between the CK and Sm-treated groups. *Trichoderma* was more abundant in the control samples, whereas unclassified *Chytridiomycota* showed a higher relative abundance in the Sm-treated samples and was rarely detected in the control group (Figure 6D).

#### 2.4.3. Venn Diagram Analysis

**Figure 7:**
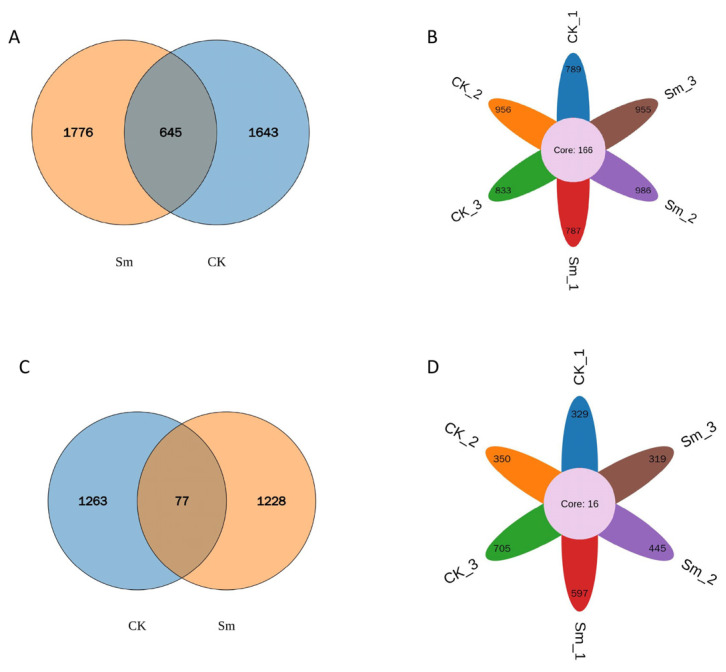
Venn diagram analysis. (**A**) Venn diagram of bacterial OTUs/ASVs; (**B**) Core OTU/ASV analysis of bacteria; (**C**) Venn diagram of fungal OTUs/ASVs; (**D**) Core OTU/ASV analysis of fungi. Numbers in the plots indicate the observed number of OTUs/ASVs in the corresponding group or intersection. “Core” represents the taxa present in all samples.

Among all detected bacterial taxa, 1776 features were unique to the Sm-treated group, 1643 were unique to the CK group, and 645 were shared between the two groups, accounting for approximately 17.5% of the total (Figure 7A). The Upset plot further quantified the feature intersections among the subgroups (CK_1/2/3 and Sm_1/2/3). A core set of 166 features shared by all subgroups was identified, indicating stable and reproducible shared features between CK and Sm groups. Meanwhile, each subgroup exhibited unique features (e.g., 789 for CK_1 and 787 for Sm_1) (Figure 7B), reflecting subgroup-specific heterogeneity. In contrast, the number of shared fungal features between the two groups was markedly reduced in this analysis, with only 77 features, accounting for approximately 2.9% of the total. The CK group had 1263 unique features, while the Sm-treated group had 1228 unique features. This distribution pattern indicates that, in this dataset, the molecular profiles of the two groups differ more markedly, with most features being condition-specific (Figure 7C). The fungal core set was greatly reduced, containing only 16 features, confirming that shared features across all subgroups were very limited. Most features were restricted to individual subgroups (e.g., 329 for CK_1 and 597 for Sm_1) (Figure 7D), consistent with the low overlap observed in the Venn diagram shown in Figure 7C.

## 3. Discussion

Previous studies have demonstrated that *S. marcescens* can function as a PGPR and exert beneficial effects in various crop systems [20]. For example, the LYGN1 strain promoted the growth of cucumber and pepper seedlings while reshaping the rhizosphere microbial community structure [21]; similarly, the UENF-22GI strain, isolated from compost, significantly enhanced the growth and the biomass accumulation of maize seedlings [7]. However, these studies have primarily focused on long-duration crops, and the growth-promoting effects in short-cycle leafy vegetables remain poorly evaluated. Moreover, most reported growth-promoting *S. marcescens* strains are derived from soil or plant tissues, and the ecological adaptability and application potential of insect-derived strains in vegetable systems remain largely unexplored and unverified.

Against this background, this study systematically demonstrated that the insect-derived *S. marcescens* can consistently exert growth-promoting effects in romaine lettuce, a short-cycle leafy vegetable system. This strain not only significantly promoted plant height, root development, and biomass accumulation but also did not dilute nutritional quality. Instead, it simultaneously increased the contents of vitamin C and total sugars in the leaves, challenging the conventional expectation that rapid growth in short-cycle plants leads to a decline in nutritional quality [22,23]. In addition, Sm treatment significantly altered endogenous hormone levels and reshaped the rhizosphere microbial community structure. These findings indicate that the growth-promoting effects of Sm are not limited to morphological improvements but may involve coordinated regulation of plant carbon assimilation and metabolic networks, thereby enhancing both primary and secondary metabolic activities.

Previous studies have shown that certain PGPR strains can exert growth-promoting effects in short-cycle leafy vegetables. For example, strains of *Bacillus* [24], *Pseudomonas*, and *Azospirillum* [25] have been reported to increase plant height, leaf area, and fresh weight of lettuce, with their growth-promoting effects being observable within a relatively short growth period. These studies indicate that the growth-promoting effects of microorganisms are not limited to long-duration crops but are also applicable in short-cycle production systems. However, compared with the more extensively studied typical PGPR groups, *S. marcescens*, although demonstrated to have growth-promoting potential, remains less investigated, and its efficacy and stability in short-cycle leafy vegetables remain poorly evaluated. The results of this study indicate that insect-derived *S. marcescens* can also significantly promote plant growth and biomass accumulation in romaine lettuce, a short-cycle leafy vegetable. This finding not only supplements the evidence for the growth-promoting potential of *S. marcescens* in short-cycle leafy vegetables but also expands the range of PGPR sources available for use in these systems.

Short-cycle leafy vegetables are typically cultivated with the primary goal of rapidly achieving marketable yield, and enhanced growth is often accompanied by preferential allocation of carbon to biomass, which can lead to a “dilution” of nutritional quality, resulting in the typical growth–quality trade-off [22,23]. However, previous studies have shown that specific strains can simultaneously improve both plant growth and quality. For example, inoculation with *Bacillus subtilis* has been reported to increase both dry biomass and leaf antioxidant content in tomato, okra, and African spinach [26]; similarly, treatment of lettuce with *Pseudomonas fluorescens* not only promoted plant height and fresh weight but also enhanced leaf total phenol and soluble sugar contents [27]. These studies show that the application of certain strains can partially overcome the traditional growth–quality trade-off, improving both plant growth and nutritional quality. In line with this, our results show that Sm treatment significantly increased plant height, root development, and biomass, while also increasing the contents of vitamin C and total sugar in the leaves. This finding confirms the growth-promoting potential of Sm in short-cycle leafy vegetables and provides a potential biostimulant strategy for short-cycle vegetable production.

Plant hormones are central signaling molecules in the regulation of plant growth, and hormone signaling interactions between PGPR and their host plants are considered to be an important component of their growth-promoting mechanisms [28]. Among these, GA and IAA are considered key hormones regulating cell elongation, vascular tissue differentiation, and root system architecture [29,30,31], whereas ABA plays a central role in carbon metabolism regulation, rhizosphere signal integration, and stress responses [32,33,34]. In this study, multiple hormone levels increased simultaneously. Although the causal order is unclear, this suggests that Sm’s growth-promoting effects are not driven by a single hormone pathway. Instead, they likely involve the coordinated regulation of several hormone signals. This multi-hormone response may be an important physiological basis for the strain to achieve rapid and stable growth-promoting effects in short-cycle leafy vegetable systems.

Numerous studies have shown that PGPR can not only directly affect plant physiology but also promote plant growth by reshaping the composition and function of the rhizosphere microbial community. For example, Zhao et al. used metagenomic analysis to show that inoculation with exogenous PGPR significantly changed the composition, abundance, and diversity of the rhizosphere microbial community in hybrid foxtail millet, and also affected key metabolic functions, including carbohydrate metabolism, energy metabolism, and signal transduction [35]. These findings indicate that the restructuring of microbial community composition and function is an important aspect of PGPR-mediated growth promotion. In addition, comparative studies of rhizosphere communities have found that plant growth performance is closely associated with the structural characteristics of their rhizosphere microbiota. For example, dominant genera such as *Arthrobacter* and *Massilia* tend to be enriched in the rhizosphere of high-yielding plants and are more closely linked to plant growth-promoting functional networks [36]. This provides community-level evidence supporting the importance of microbial restructuring for plant growth. Similar to the studies mentioned above, Sm treatment in this study significantly reshaped the rhizosphere microbial community of romaine lettuce, increasing the abundance of potential growth-promoting microbes, including *Bacillus*, *Burkholderia*, *Caballeronia*, and *Paraburkholderia*. This likely enhanced functional networks related to plant growth and ultimately promoted overall plant development.

This study systematically showed that insect-derived *S. marcescens* can promote growth, improve nutritional quality, and reshape the rhizosphere microbial community in short-cycle leafy vegetables. However, several related issues still need further investigation. First, the specific mechanisms by which this strain regulates plant hormones and optimizes metabolism need further investigation. In addition, its colonization dynamics in the rhizosphere and interactions with the native microbial community remain to be clarified. In the future, we will further explore its molecular and ecological mechanisms to provide a stronger basis for the efficient application of this strain. In summary, this study systematically verified the growth-promoting effects of insect-derived *S. marcescens* in a short-cycle leafy vegetable system from three aspects: growth traits, nutritional metabolism and endogenous hormone levels, and rhizosphere microecological restructuring. These results expand the applicable scope of the growth-promoting function of *S. marcescens* in vegetable production systems. They also highlight the important potential of insect-associated microbial resources for the development of functional agricultural microorganisms. This provides new microbiological support for the efficient and sustainable production of short-cycle leafy vegetables.

## 4. Materials and Methods

### 4.1. Strain Source

The bacterial strain *S. marcescens* BRC-CXG2 used in this study was isolated from a naturally deceased fourth-instar larva of *Monochamus alternatus*. The strain is currently preserved at the Key Laboratory of Major Pest Control in Ecological Public Welfare Forests of Fujian Province, Fujian Agriculture and Forestry University, China.

### 4.2. Experimental Materials and Culture Conditions

The tested plant was romaine lettuce, cultivar ‘Sijiqing Youmaicai’. The seeds were obtained from Qingxian Xingyun Seed Industry Co., Ltd. (Qingxian, China). The seeds were surface-sterilized with 75% ethanol and rinsed three times with sterile distilled water. Subsequently, the sterilized seeds were sown in seedling trays filled with a sterilized peat-based nursery substrate, with 1–2 seeds per cell, and covered with a thin layer of substrate. The trays were placed in an incubator to allow germination. After germination, uniform seedlings were selected and transplanted into plastic pots (approximately 10 cm in diameter) containing a substrate mixture of peat and perlite (3:1, *v*/*v*). The plants were cultivated in a growth chamber at 20 °C and 70% relative humidity under a 16 h light/8 h dark photoperiod, with a light intensity of 200 μmol·m^−2^·s^−1^.

### 4.3. Strain Treatment and Inoculation Method

*S. marcescens* was grown in LB liquid medium at 28 °C and 200 rpm until the logarithmic phase. The cells were collected by centrifugation (8000 rpm for 8 min) and resuspended in sterile distilled water. The bacterial concentration was estimated by measuring OD_600_ using a spectrophotometer (UV-1800, Shimadzu Corporation, Kyoto, Japan), and adjusted to approximately 1 × 10^8^ CFU·mL^−1^, a concentration optimized through repeated preliminary experiments for maximal growth-promoting effect. Romaine lettuce seedlings at 7 days after emergence were inoculated by the root-drenching method, with 50 mL of bacterial suspension applied per plant. The control group received an equal volume of sterile distilled water. The number of biological replicates (*n*) varied depending on the experiment and is indicated in the corresponding figure legends.

### 4.4. Plant Growth and Biomass Measurements

Samples were collected one month after treatment. Plant height, root length, and other growth parameters were measured using a ruler. The harvested plants were separated into aboveground and belowground parts, and fresh weights were determined using an electronic balance. Dry weights were measured after drying at 65 °C until constant weight.

### 4.5. Leaf Nutritional Quality

Samples were collected one month after treatment, quickly frozen in liquid nitrogen, and stored at −80 °C for further analysis.

#### 4.5.1. Determination of Vitamin C Content

The vitamin C content in romaine lettuce leaves was determined using the 2,6-dichlorophenol-indophenol (DCPIP) titration method [37]. One gram of fresh leaf tissue was weighed and ground with 2% (*w*/*v*) oxalic acid solution for extraction. The mixture was thoroughly homogenized at low temperature and then filtered, and the filtrate was collected as the test solution. The filtrate was titrated with a prepared DCPIP solution (Solarbio, Beijing, China). The endpoint was reached when a light red color appeared and persisted for 30 s without fading. The vitamin C content in the sample was calculated based on the volume of DCPIP solution consumed during titration and expressed as a percentage.

#### 4.5.2. Determination of Total Sugar Content

The total sugar content in the leaves was determined using the anthrone–sulfuric acid colorimetric method [38]. One gram of fresh leaf tissue was weighed and ground with distilled water. The homogenate was then boiled in a water bath for 10 min and filtered through filter paper to collect the extract for further analysis. An aliquot of the filtrate was mixed with anthrone reagent (Solarbio, Beijing, China) and concentrated sulfuric acid. After reaction in a boiling water bath for 10 min, the absorbance was measured at 620 nm using a UV-visible spectrophotometer (UV-1800, Shimadzu Corporation, Kyoto, Japan). The total sugar content of the samples was calculated using a glucose standard curve and expressed as a percentage.

#### 4.5.3. Determination of Chlorophyll Content

One gram of fresh leaf tissue was weighed and ground with 80% acetone in the dark to fully extract the leaf pigments. After standing, the supernatant was collected for analysis. Absorbance was measured at 663 nm and 645 nm using a spectrophotometer (UV-1800, Shimadzu Corporation, Kyoto, Japan), and chlorophyll a, chlorophyll b, and total chlorophyll contents were calculated according to the classical formulas established by Arnon [39]. The results were expressed in mg·g^−1^.

### 4.6. Determination of Endogenous Hormones

The endogenous hormone levels, including IAA, GA_3_, and ABA, were determined using enzyme-linked immunosorbent assay (ELISA) kits (IAA: MM-0953O1; GA: MM-0125O1; ABA: MM-0138O1; Meimian, Qinzhou, China) according to the manufacturer’s instructions. The antibody specificity and cross-reactivity of the ELISA kits are not fully characterized by the manufacturer, and therefore the measured values represent total immunoreactive hormone equivalents rather than specific bioactive forms.

Frozen plant samples (1 g) were ground thoroughly in liquid nitrogen and homogenized with 9 mL phosphate-buffered saline (PBS). The homogenate was transferred into centrifuge tubes and centrifuged at 3000 rpm for 20 min at 4 °C using a refrigerated centrifuge (5810R, Eppendorf, Hamburg, Germany). The supernatant was collected for subsequent hormone analysis.

Hormone concentrations were determined based on antibody–antigen reaction with horseradish peroxidase (HRP)-mediated colorimetric detection. Briefly, standards and sample extracts were added to microplate wells and incubated according to the kit protocol. After washing to remove unbound substances, tetramethylbenzidine (TMB) substrate solution was added for color development. The reaction was terminated by adding stop solution, resulting in a color change from blue to yellow. The absorbance was measured at 450 nm using a microplate reader, and hormone levels were calculated based on the corresponding standard curves. Hormone levels were expressed as GA_3_-immunoreactive equivalents (ng g^−1^ FW) and IAA- and ABA-immunoreactive equivalents (μg g^−1^ FW). All measurements were performed with three biological replicates.

### 4.7. Rhizosphere Microbial Community Analysis

Rhizosphere soil samples were collected from the CK and Sm treatments, with three biological replicates per treatment. During sampling, loosely attached soil was gently shaken off, and the soil adhering closely to the roots was carefully scraped into sterile tubes. After collection, the samples were immediately frozen in liquid nitrogen or on dry ice and then quickly transferred to −80 °C for storage to preserve the rhizosphere microbial composition.

#### 4.7.1. DNA Extraction and Amplification Sequencing

Total genomic DNA was extracted from 0.5 g of fresh soil using the MagBead-based Environmental Microbial Genomic DNA Extraction Kit (Cat. No. DP713; Tiangen Biotech, Beijing, China), according to the manufacturer’s instructions. DNA integrity was assessed by 1% agarose gel electrophoresis, and DNA concentration and purity were measured with a Qubit fluorometer (Thermo Fisher Scientific, Waltham, MA, USA). The V3–V4 region of the bacterial 16S rDNA gene was amplified using primers 341F (CCTACGGGAGGCAGCAG) and 805R (GACTACHVGGGTATCTAATCC), while the fungal internal transcribed spacer 1 (ITS1) region was amplified using primers ITS1FI2 (GAACCWGCGGARGGATCA) and ITS2 (GCTGCGTTCTTCATCGATGC). PCR amplification, library construction, and sequencing were performed by a commercial sequencing service provider, with Illumina adapter overhang sequences added to the primers for library preparation, and sequencing was conducted on the Illumina NovaSeq 2500 platform (Illumina, San Diego, CA, USA) (paired-end 250 bp).

The paired-end reads obtained from sequencing were assigned to their respective samples based on sample barcodes. Adapters and primer sequences were removed, followed by quality control and chimera filtering. High-quality sequences were denoised using DADA2 to generate an ASV table, and all subsequent diversity and abundance analyses were based on the ASV data. To ensure comparability between samples, the sequence counts were rarefied and normalized.

#### 4.7.2. Microbial Diversity Analysis

Principal coordinate analysis (PCoA) was performed based on the ASV table to visualize microbial community structure and to examine clustering and separation among groups. The relative abundances of microbial communities were analyzed at different taxonomic levels (phylum/genus) to compare changes in dominant taxa between treatment groups. Venn diagrams were used to assess group-specific and shared ASVs, and the number of core and group-specific ASVs was quantified. Microbial sequences were aligned and taxonomically annotated using the SILVA database for bacteria and the UNITE database for fungi, with BLAST + v2.17.0 employed for verification.

### 4.8. Statistical Analysis

Differences in physiological parameters, nutritional components, and endogenous hormone levels between groups were assessed using independent-samples (unpaired) *t*-tests using GraphPad Prism 8.0. Microbial community analyses were based on ASV data. Core sequences present in all samples and group-specific sequences were identified. UpSet plots were used to compare feature distributions among different treatment groups.

## 5. Conclusions

To our knowledge, this study is the first to confirm that insect-derived *S. marcescens*, isolated from a deceased *M. alternatus* larva, can promote the growth of romaine lettuce. The experimental results indicated that Sm treatment significantly increased plant height, root length, and both aboveground and belowground biomass. In addition, the contents of vitamin C and total sugars in leaves were significantly increased, and the levels of GA_3_-, ABA-, and IAA-immunoreactive equivalents were also elevated. Microbial community analysis showed that Sm application altered the structure of the rhizosphere microbial community, resulting in shifts in the relative abundance of specific bacterial and fungal taxa, indicating a regulatory effect on community composition. Overall, these findings demonstrate that insect-derived *S. marcescens* can promote plant growth, improve nutritional quality, and modulate rhizosphere microecology in short-cycle leafy vegetable systems, thereby providing experimental evidence and a theoretical basis for the application of such functional strains in vegetable production.

## Data Availability

The original contributions presented in this study are included in the article. Further inquiries can be directed to the corresponding author.
